# Optimizing the Agricultural Supply Chain through E-Commerce: A Case Study of Tudouec in Inner Mongolia, China

**DOI:** 10.3390/ijerph20053775

**Published:** 2023-02-21

**Authors:** Jian Li, Xin Yan, Yongwu Li, Xuefan Dong

**Affiliations:** College of Economics and Management, Beijing University of Technology, Beijing 100124, China

**Keywords:** rural e-commerce, rural revitalization, agricultural supply chain, supply chain finance

## Abstract

E-commerce has the potential to address problems in the agricultural supply chain and support the implementation of rural revitalization strategies. Previous research has largely focused on the business models of rural e-commerce platforms, but has not examined the mechanisms by which they can optimize and reconfigure the agricultural supply chain. This study aims to fill this gap through a case study of Tudouec, a potato e-commerce platform in Inner Mongolia, China. The study employs a single-case study method and utilizes data from interviews, fieldwork, and secondary sources. The findings show that Tudouec is a multi-functional platform offering technical support, warehousing, logistics, supply chain finance, and insurance, among other services. It not only serves as a multi-channel information management platform, but also enhances supply chain capabilities through the interaction of information flow with capital and material flows. This rural e-commerce model addresses the limitations of traditional agricultural models and promotes poverty reduction and rural revitalization. The study’s main contribution is in demonstrating the potential for the Tudouec model to be applied to other agricultural products and in other developing countries.

## 1. Introduction

Since the implementation of the reforming and opening up policies in China, there has been a marked disparity in the rate of development between rural and urban regions [1]. Imbalanced urban–rural development, and a lack of progress in rural areas, have become major issues in contemporary Chinese society [2]. In response to these pressing rural challenges, China introduced the rural revitalization strategy in 2017, which places a particular emphasis on the growth of agriculture, rural regions, and farmers [3,4].

The Agricultural Supply Chain (ASC) is considered to play a critical role in farmers’ income, rural economic growth, social development, and environmental sustainability, as demonstrated by numerous studies [5,6]. Despite its importance, ASC management is considered to be challenging when compared to other industries. This can be attributed to a number of factors, one of which is the sensitivity of agriculture to natural influences, such as weather conditions, regional climatic differences, soil quality, and seasonal variations [7]. Additionally, agricultural markets tend to be unstable and highly susceptible to economic and financial fluctuations, including changes in market supply and demand conditions, distribution channels, and harvest periods [8]. This high level of uncertainty in agriculture poses significant risks to ASC management [9], including difficulties in Supply Chain Management (SCM), due to environmental variability [10], long chains affecting quality consistency levels of agricultural products [11], and the dependence of agricultural production on the environment, as well as high distribution costs, due to the perishable nature of agricultural products [12]. These factors can significantly impact agricultural development and farmers’ income [13].

The proliferation of information and communication technology, particularly the growing prevalence of e-commerce in developing nations, such as China, and nations in Southeast Asia and Africa, has resulted in significant improvement in rural economies [12]. Rural e-commerce is regarded as a key factor in reducing the disparity between urban and rural areas, and in boosting the income of rural dwellers [14]. Adoption of e-commerce enables small-scale farmers to overcome barriers to market access and engage in online transactions, thereby accessing both national and global markets [15]. The elimination of price squeeze and information asymmetry by intermediaries has enabled the farmers to sell their products at higher prices compared to before [9]. E-commerce has been shown to enhance farm performance and decrease transaction costs [16]. Agricultural e-commerce in China has seen tremendous growth, owing to continued high-level support and promotion from the central government [9,17]. According to data from the Ministry of Commerce (2020), rural online retail sales in China reached 1.7 trillion yuan in 2019, accounting for 16.1% of total retail sales, with a growth rate of 19.1% which was 2.6% higher than the growth rate of total retail sales. Online retail sales of agricultural products reached 397.5 billion yuan in the same year. Therefore, exploring the implementation of e-commerce in ASC could play a role in advancing rural revitalization.

An ASC refers to the series of production-to-market activities involved in the transportation of agricultural products from the farm to the consumer’s table [18]. This encompasses all processes, from production on the farm to processing, distribution, and retailing, ultimately reaching the end-user [8]. In the academic sphere, numerous studies have compared ASCs with other supply chains so as to identify the defining characteristics of ASCs. Like other supply chains, ASCs are an intricate network of organizational entities that bring products and services to the market with the aim of satisfying the needs of customers [19]. Despite being closely akin to fast-moving consumer goods supply chains in many aspects, the primary difference is that ASCs source raw materials directly from fields, with the final products intended for either human or animal consumption [20]. The objective of ASC management is to swiftly and efficiently transport agricultural products from farmers to end-users, while minimizing damage to the products [21]. This not only contributes to the financial stability of farmers by ensuring fair returns, but also ensures that consumers receive high-quality products. However, the unique characteristics of agricultural products, such as limited shelf life, variability in demand and price, and consumer requirements for product traceability, make ASCs more complex and challenging to manage, compared to other supply chains [22]. Consequently, it can be challenging to apply the practical experience of SCM from other industries to the management of ASCs [23]. Given the critical role that product traceability, quality specifications, and food safety play in ASCs, effective ASC management necessitates the incorporation of activities and decision-making processes at the strategic, tactical, and operational levels [5,24]. Consequently, this research aimed to examine the potential for e-commerce to optimize the agricultural supply chain by restructuring three flows (material flow, information flow, and financial flow [25]) of the supply chain, in accordance with the unique characteristics of the agricultural supply chain.

Despite the challenges posed by the inherent characteristics of agricultural products to the implementation of e-commerce in agriculture, there remains a positive outlook towards its future application, as demonstrated by several studies [26,27,28,29,30]. The application of rural e-commerce has been touted as a means of transforming the configuration and relationships between various segments of the ASC [31,32]. The potential benefits of rural e-commerce include reduced production and transaction costs, improved logistics and distribution efficiency, decreased information asymmetries, enhanced links between agricultural production supply and demand, and facilitated connections between users globally [33,34,35]. Li et al. [36], Zhu et al. [37] and Juan et al. [38] also found that rural e-commerce offers significant advantages in enhancing the marketing and trading of agricultural products. Chiang et al. [39] were the first to propose the concept of rural e-commerce for supply chain optimization and found that its adoption into the traditional agricultural channel could result in improved ASC efficiency. Fritz et al. [31] posited that rural e-commerce involves the utilization of electronic strategies in interaction and transaction between participants in the agricultural industry, leading to new relationships and reconfigured relationships between various stages and segments of ASCs. Research into the optimization of the supply chain by e-commerce mainly focuses on its intermediary and information-sharing roles [30]. Li et al. [32] found that e-commerce enables the creation of effective information sharing mechanisms within an ASC. Zeng et al. [9] believed that rural e-commerce could mitigate the negative effects of information asymmetry caused by physical distance and increase the selling prices for small farmers. GuoHua et al. [40] used an evolutionary game model to compare traditional and modern ASCs and found that e-commerce could address the problem of information asymmetry. However, there are also opposing viewpoints among scholars. Some limitations and challenges in the implementation of e-commerce in ASCs have been brought to light. Mueller [14] analyzed the impact of e-commerce on the agricultural market and concluded that existing e-commerce for agricultural products suffers from low logistics efficiency, high costs, and low service levels, which cannot meet consumers’ requirements for high quality and efficiency. Zhao et al. [41] also identified some problems with national rural e-commerce platforms and suggested that local e-commerce platforms could be more efficient in ASC management. Bao et al. [42] and Montealegre et al. [43] pointed out that the deep integration of e-commerce and ASC still requires further analysis of the supply chain process. However, few studies have focused on how rural e-commerce platforms optimize and reconstruct ASCs, and few studies have analyzed their operational mechanisms by examining the three flows of the supply chain [9].

Numerous scholars have investigated the mechanism of supply chain optimization by examining the interplay between the three flows [44,45]. Kim et al. [46] posited that the management of these flows constitutes the foundational components of e-commerce supply chain management activities. Studies have been conducted on the interdependence between the three flows, exploring how they can be integrated to deliver more efficient supply chain services. Sahin et al. [47] and Lee et al. [48] emphasized that, without information flow, material and financial flows are unable to function optimally. Rai et al. [49] suggested that information technology could significantly improve supply chain performance by integrating information and material flow. Costa et al. [50] found that the integration of information and material flows through RFID technology contributed to quality management of agricultural products. The integration of capital flow and logistics could enhance inventory management and financial planning [51], as demonstrated by studies, such as those by Pfohl et al. [52] and Wang et al. [53], showing that the segmentation and integration of the three flows could reflect the underlying operational mechanisms of the supply chain and improve its service capability through effective integration.

In light of these findings, this study aimed to shed light on how rural e-commerce platforms can construct a comprehensive and integrated agricultural supply chain model. The study examines the relationship between rural e-commerce platforms and the supply chain by investigating the enhancement, interaction, and integration of information flow, capital flow, and logistics. Through a case study approach, the study aimed to distil a model of rural e-commerce platforms and the agricultural supply chain that can be extrapolated and applied to other regions and agricultural products to advance agricultural development.

## 2. Materials and Methods

### 2.1. Methodology

The utilization of a qualitative case study approach was deemed appropriate for this research in order to explore the unique phenomena and questions present within the complex background. The case study method, as discussed by Yin et al. [54], is suitable for studying the formation of rare and significant emerging phenomena that involve diverse background conditions, multiple sources of evidence, and dynamic changes. The selection of Tudouec as the subject for a single case study was based on two factors. Firstly, as highlighted by Zhao et al. [41], local e-commerce platforms exhibit greater capability in mobilizing resources and promoting agriculture compared to national agricultural e-commerce platforms. Hence, it was deemed more appropriate to choose a representative local e-commerce platform for a single case study. Secondly, there is limited research on the integration of the three flows in rural e-commerce supply chains, making Tudouec an ideal candidate, as it represents a noteworthy example of this integration and is, therefore, deserving of an in-depth study.

### 2.2. Case Selection

**Figure 1:**
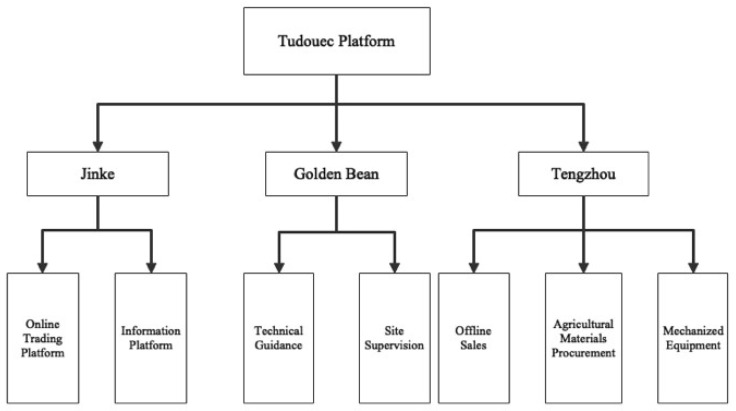
Organizational chart and departmental mission chart of Tudouec.

Inner Mongolia is China’s largest potato production base, where production accounts for about 1/7 of the national production [55]. The region is characterized by abundant light, large diurnal temperature differences, and light soil texture, which are favorable for potato production [56,57]. Tudouec E-commerce Platfrom (Tudouec) was founded in 2012 in Hohhot, Inner Mongolia, China. Unlike other rural e-commerce platforms, Tudouec not only has the function of collecting, processing, and releasing information, but also breaks the information asymmetry and takes the initiative to optimize the supply chain to build a virtuous agricultural benefit ecosystem. The platform effectively integrates supply, production, processing, sales, storage, and logistics. Table 1 and Figure 1 show the composition of the Tudouec Platform and organizational chart. The platform consists of three subsidiaries, each with different functions. Specifically, Jinke is responsible for the operation of the online trading platform and the construction of the information platform. Golden Bean is stationed with account managers and technicians to provide offline agricultural planting technological guidance and supervision for farmers. Tengzhou handles the procurement of agricultural materials, such as seeds and chemical fertilizers. It also provides mechanized equipment rental services and offline distribution sales services for farmers.

Compared with Alibaba and JD.com, the Tudouec platform is only a small platform for regional and niche agricultural products categories. However, the continuous growth of the Tudouec platform’s trading volume for several years in a row proves its superiority to some extent. Moreover, it is a rare comprehensive rural e-commerce platform that combines the platform business model, SCM model, and supply chain finance model. Therefore, it is a representative case study that deserves to be studied in depth.

### 2.3. Data Collection and Analysis

The data used in this study were obtained from both primary and secondary sources and were subjected to triangulation to ensure the reliability and validity of the results. Triangulation is recognized as an essential method in case study research [54]. This study mainly collected data from five sources: interviews, participatory observation, official websites, cooperative financial institutions, and media reports. The utilization of multiple data sources allowed for triangulation [58], thereby enhancing the credibility and accuracy of the study’s conclusions. The primary data were mainly obtained by the following means:

Interviews: To obtain the primary data needed, semi-structured interviews and focus group interviews were conducted in this research. Semi-structured interviews are not only highly maneuverable but also allow the interviewers to engage in in-depth communication with the interviewees. As shown in Table 2, a total of 34 interviews were conducted for relevant individuals in this study. The interviews were led by one lead interviewer, who was a native speaker of the local language, and the average length was about one hour. Focus group interviews were also conducted in this research to ensure that all the participants expressed their views and to avoid the dominance of particular individuals and ensure group conformity [59]. Most respondents were interviewed more than once for this study in order to obtain comprehensive information and to ensure the authenticity of the information conveyed. The initial interviews and investigation were conducted on 17–24 May, 2020, and the supplemental interviews were carried out on 13–14 July, 2020. To reveal the changes in the villagers’ income structures, we also interviewed five rural villagers who were not involved in Tudouec. The question designed for this group only focused on their planting and marketing model.

The data collection for this study involved a combination of qualitative research methods, including in-depth interviews, participatory observation, and secondary information collection. The interviews were conducted with key stakeholders of the case company and covered six essential aspects of the company’s operation: business model, distribution model, information management model, supply chain finance model, and SCM model. The interviews were recorded and transcribed, resulting in 100 pages of transcripts.

The interviews were composed of the following six parts to gain necessary information about the company: overall operation and business model, distribution model, information management model, supply chain finance model, and SCM model. The interviews were recorded and transcribed, resulting in 108 pages of transcripts. Participatory observation: field visits to the sample enterprises focused on understanding the specific mode of operation of Tudouec and the SCM methods. This further enriched the study’s research data.

Secondary information was obtained mainly through online articles, news, reports, and videos. An example was Tudouec platform information, which included the following: (1) the official website and WeChat official account; (2) cooperative financial institutions; (3) tracking media reports.

The collected data underwent coding and analysis through the utilization of Microsoft Excel for data reduction and coding. Participatory research and interviews with the principals of the three companies under the Tudou platform were conducted to examine their historical background, development, and scope of business. The framework of the companies was constructed (referred to in Figure 1), taking into account their respective roles and positions within the platform. This served to better illustrate the organizational structure of the companies. Building on this initial step, the network structure of the platform was drawn, based on the companies’ framework, in order to categorize the functional aspects of the platform. The supply chain services provided by Tudouec were vertically divided into e-commerce trade, offline services, and finance-related services. To further study the underlying mechanisms of its supply chain management, the supply chain model of Tudouec was mapped, based on the information flow, financial flow, and material flow, and the findings from the previous steps integrated with, and incorporating, data obtained from the semi-structured interviews. Finally, the accuracy of the design was verified through mutual validation with the participants of the platform, confirming the validity of the results of the case study.

## 3. Case Description

### 3.1. Traditional ASC Model and Existing Problems

**Figure 2:**
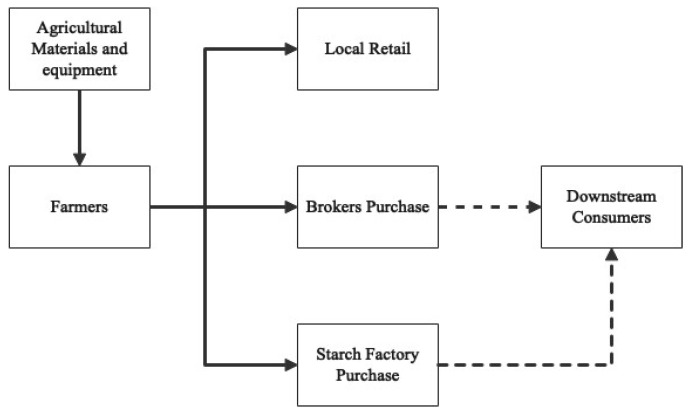
Traditional ASC model.

As depicted in Figure 2, a traditional ASC is characterized by its simplicity and fragility. The distribution channels available to farmers, including local retail, broker purchase, and starch factory purchase, are limited in scope [40,60]. While local retail offers higher unit prices, it is characterized by lower sales volume and stringent product quality requirements. Starch factories possess significant bargaining power and are selective in the varieties they purchase. Unlike the traditional ASC, brokers exploit information asymmetry and transportation bargaining power, leading to a substantial reduction in farmers’ profits. The dotted line in the figure linking only the middlemen and starch factories with downstream consumers highlights the difficulty that farmers face in directly connecting with their customers.

In sum, the problems of traditional ASCs are mainly focused on market channels, information access, cultivation, finance, insurance, logistics, and storage (Table 3). First, the large number of farmers, the small scale of single-family farming, and the low degree of organization lead to farmer groups failing to establish a scale effect in the market. The unit transaction cost of almost all transactions is high [13]. Second, market imperfections are prevalent in developing countries, such as lack of technology and price opacity, weak linkages to downstream markets, and credit constraints [61]. Third, the poor efficiency of information flow, the lack of financial and insurance services, the incomplete and expensive logistics system, and the inferior storage conditions all constrain the development of the rural economy.

### 3.2. Description of the Tudouec Business Model

**Figure 3:**
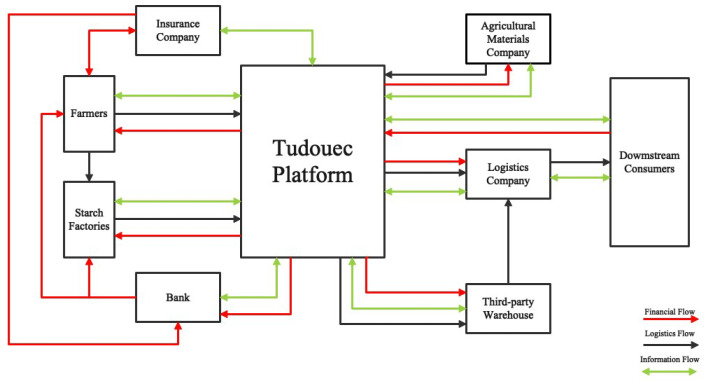
Supply chain model of Tudouec.

Traditional ASCs needs to be integrated and redesigned to make them more efficient and competitive [8]. As shown in Figure 3, Tudouec generally weaves a dense supply chain network that connects farmers, starch factories, insurance companies, financial institutions, warehouse companies, logistic companies, and downstream customers together. Compared with the traditional ASC model, this model looks complex and bloated. However, complexity does not mean inefficiency. Rather, Tudouec creates a full-function supply chain system. Tudouec acts as an integrator of information to guide the development of agricultural production plans by analyzing the overall supply and demand levels of the industry in real time. In addition, Tudouec acts as an online trade exchange platform, which improves agricultural products’ distribution efficiency and increases the profit margin. Tudouec also has the function of providing quality agricultural material supplements, including seedlings, pesticides, fertilizers, machinery, accessories, crop health care, and guidance in planting techniques. Logistics, warehousing, and cold storage services are also included. Financing and insurance are also available for members who join the platform. Therefore, we provide an in-depth analysis of the emergence mechanism of this business model.

**Figure 4:**
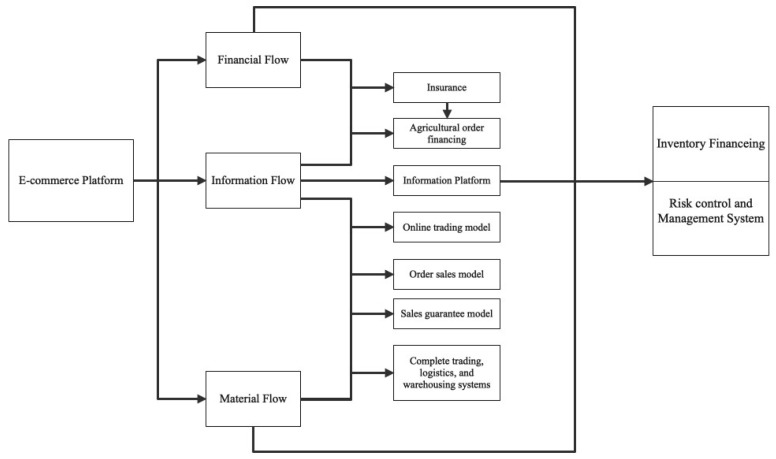
Research Roadmap.

We established a research road map, depicted in Figure 4, to shed light on the functioning of the Tudouec platform and the integration of the three flows that drive the generation of SCM tools. Our initial focus was on the significance of the information flow. By considering the information flow as the core of Tudouec, we aimed to examine how the platform integrates data from all participants to establish a well-functioning agricultural supply chain network, thereby overcoming the challenges of information silos commonly encountered in traditional supply chains. Next, we analyzed the integration of the information flow with the capital flow and its potential to generate supply chain insurance and financial instruments. Furthermore, we studied the integration of the information and material flows and its impact on the development of new sales models, logistics, and warehousing strategies. Our aim was to provide a complete understanding of the various techniques and strategies employed to achieve this integration and the benefits yielded. Finally, we delved into the interplay of the three flows, leading to the optimization of the supply chain. Our analysis shed light on the capabilities generated by the combined efforts of the information, capital, and material flows. The ultimate objective of this discussion was to provide a comprehensive understanding of the operational mechanism of the Tudouec platform and how it was designed to optimize and reconfigure the agricultural supply chain.

## 4. Discussion

### 4.1. Capabilities Generated from Information Flow

Information sharing is a crucial component of successful SCM [62,63]. This sharing helps coordinate business processes and improve services provided to customers [53]. Studies have consistently demonstrated that information flow is essential to a functioning supply chain and takes precedence over other types of flows [62,64]. Information and Communication Technology (ICT) play a vital role in the agribusiness sector, as they can enhance the efficiency, sustainability, flexibility, and resilience of the entire supply chain from farmer to end customer [65]. Despite rapid advancements in ICT, rural areas continue to lag behind urban areas, especially in developing countries, where information asymmetry is prevalent across all stages of the Agricultural Supply Chain (ASC), resulting in reduced supply chain efficiency.

**Figure 5:**
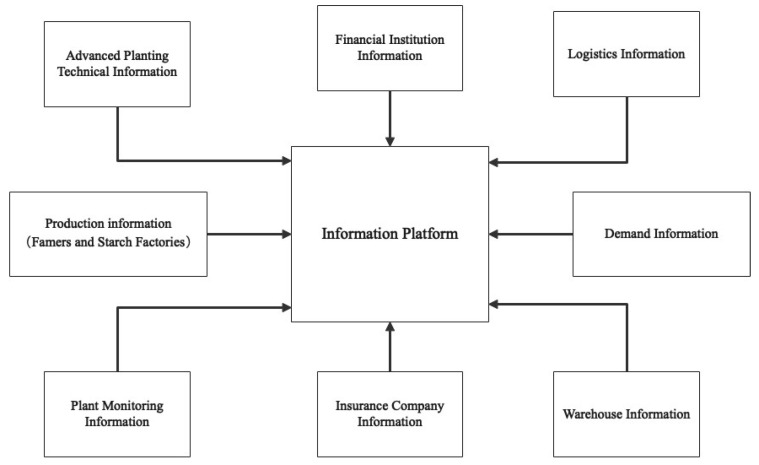
Information integration model of Tudouec.

**Figure 6:**
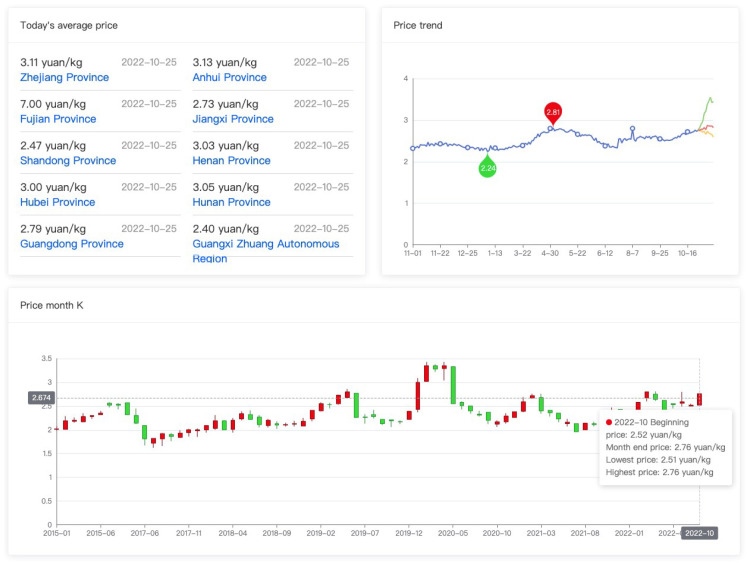
Monthly K chart of the national wholesale potato market prices (www.tudouec.com, accessded on 25 October 2022.).

The Tudouec platform aims to collect, analyze, publish, and exchange information. As depicted in Figure 5, the platform creates a diverse and complex information network that allows each participant to access and aggregate information, thereby establishing an efficient and transparent information exchange channel. Firstly, the platform facilitates an efficient and transparent information exchange channel to collect and summarize supply and demand information for the industry as a whole. Through big data analysis, the platform matches buyers and sellers with accuracy. As illustrated in Figure 6, analysis of price change trends can help farmers make informed decisions about the best-selling price for their products. The platform also predicts market demand, enabling farmers to make informed decisions about the variety and scale of crops to plant the following year. Secondly, the platform provides farmers with access to advanced farming techniques through regular visits from technicians who teach and monitor the farming process. The platform leverages its information and scale advantages to negotiate competitive prices for high-quality seeds, fertilizers, and agricultural machinery with suppliers. Lastly, the platform implements cameras and internet of things monitoring equipment in plantation areas to monitor crop growth in real-time. Information about planting conditions is uploaded to the platform, making it accessible to all participants. In the future, the platform intends to utilize blockchain technology to provide traceability functions [66].

### 4.2. Capabilities Generated from Information Flow + Financial Flow

#### 4.2.1. Insurance

**Figure 7:**
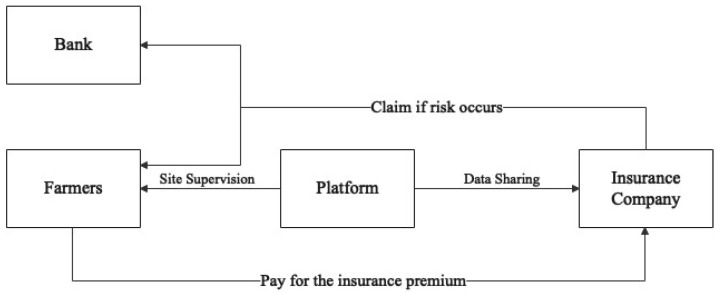
Platform insurance model.

The provision of insurance services by insurance companies to eligible farmers in the Tudouec platform is depicted in Figure 7. In this partnership, farmers are able to access insurance coverage at a reduced premium rate of 3%, which is lower that of traditional insurance products offered outside of the platform. The insurance coverage compensates for up to 90% of losses incurred due to natural disasters or low market prices. The effective management of information flow is a key aspect of this arrangement as Tudouec shares relevant data, including planting information, order information, and market information with the insurance companies. This helps to bridge the information gap that often exists in the traditional insurance industry. Additionally, the platform’s account managers, who provide guidance and supervision of the planting process, serve as a crucial reference for the insurance companies in reducing risks and promoting effective risk management practices [67].

#### 4.2.2. Agricultural Order Financing

**Figure 8:**
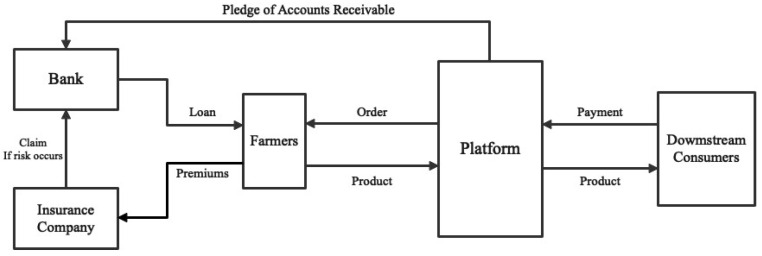
“Platform + Insurance + Order Financing” Model.

The lack of a stable repayment source, collateral, and low credit rating have long posed challenges for farmers in securing adequate financing for their operations. Despite the need for capital to expand and reproduce, traditional financing options for farmers are often difficult and expensive to obtain [68]. Supply chain finance, which leverages the combination of information flow and capital flow resources, has emerged as a solution to this problem. Reliable information can be used to mitigate investment risk within the supply chain and reduce the cost of capital [52]. Tudouec’s platform, which is built upon information and logistics, serves as the cornerstone of the bank’s “platform + insurance + order financing” model. This model is designed to provide low-cost credit funds to farmers, while also addressing the risk management challenges faced by the bank. In the context of agricultural supply chains (ASCs), order farming is an upstream intervention mode that can be considered a supply chain finance scheme [69]. The order financing model, as illustrated in Figure 8, enables farmers to secure financing through the information and logistics infrastructure provided by Tudouec, thereby improving access to capital and promoting sustainable agricultural development.

The borrowing individuals in this financial model are farmers who have been active participants on the Tudouec platform for a minimum of five years and have cultivated a minimum of 33 acres in the current planting season. The financial institution, in this case a bank, leverages data sharing with the Tudouec platform to obtain information related to the scale of planting, production, and market orders. As a result, the bank is able to offer unsecured loans to potato farmers with interest rates lower than 8%. During the planting period, Tudouec provides technical support and on-site supervision to farmers, who are required to adhere strictly to the platform’s established planting standards for planting, harvesting, and disease/pest control. This information is recorded in real-time and uploaded onto the platform, providing valuable data for post-credit management and insurance supervision by financial institutions and insurance companies. Moreover, the intermediary role played by the Tudouec platform ensures that farmers receive payment for their sales, while also ensuring that downstream customers receive high-quality products. In the event of market fluctuations or product stagnation, the Tudouec-assisted starch factory promises to purchase any unsold potatoes. If the crops suffer losses due to natural disasters or low market prices, the insurance company is responsible for compensating farmers and the bank for 90% of the losses, thereby minimizing potential losses and reducing risks for all involved parties.

### 4.3. Capabilities Generated from Information Flow + Material Flow

#### 4.3.1. Complete Trading, Logistics, and Warehousing Systems

The coordination of material flow in the agricultural industry is greatly improved through the facilitation of information flow. Historically, farmers have faced challenges in obtaining reasonable prices for the rental of logistics and warehousing services due to their limited bargaining power [41]. However, as the platform continues to grow and attract a larger number of stakeholders, including farmers, service providers, and downstream customers, the emergence of economies of scale has led to an increase in the number of logistics and warehousing enterprises settling in, and, thus, enhancing the overall capacity of the supply chain. Specifically, the improvement of logistics capacity offers farmers the ability to overcome the limitations imposed by distance, reducing the loss associated with transportation and enabling long-distance and high-priced orders to be fulfilled. The enhancement of storage capacity, on the other hand, reduces storage loss and extends the sales period, contributing to higher income for farmers.

The integration of information flow and material flow is demonstrated in two distinct areas. Firstly, the application of information technology in logistics and warehousing allows for real-time monitoring of cargo status, offering greater visibility and control over the supply chain. Secondly, the platform facilitates the sharing of information regarding storage and logistics capacities, enabling individual farmers to benefit from shared resources and cost savings, effectively reducing costs and minimizing resource waste.

#### 4.3.2. Smart Plantation

Tudouec is currently exploring the potential of a technologically advanced smart plantation system. This innovative approach integrates several cutting-edge technologies, including 5G, drones, video surveillance, temperature sensors, humidity sensors, and automatic sprinkler irrigation. The deployment of these technologies has several significant benefits, including reduced labor costs, increased efficiency and quality of the planting process, and it addresses critical issues in food safety and traceability. By leveraging these advanced technologies, Tudouec aims to create a smarter, more efficient, and more sustainable agricultural ecosystem that meets the evolving needs of farmers and consumers alike.

#### 4.3.3. Reshape Agriculture Marketing

**Figure 9:**
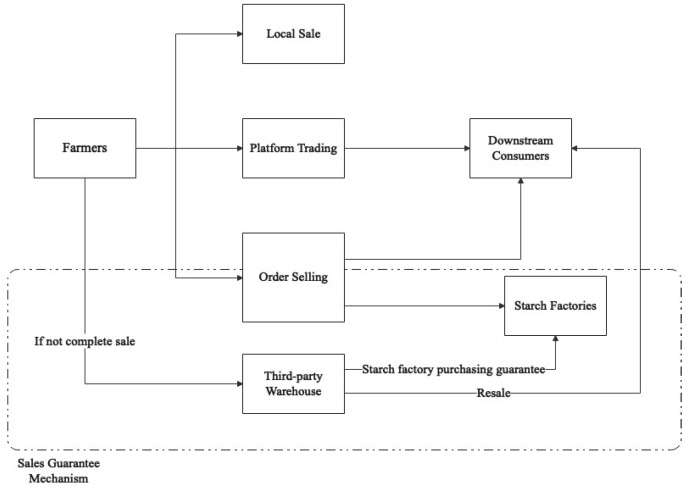
Sales model of the Tudouec platform from the perspective of farmers.

As illustrated in Figure 9, the platform challenges the conventional sales model by establishing three new models of online trading, order sale, and sales guarantee. The online trading model enables both the supply and demand sides to publish trading information and take advantage of the platform’s intermediary function for secure transactions. The order sale model, in turn, ensures steady supply and stabilizes prices through a three-step process. Firstly, the platform collects information on the production capacity of farmers, the demands of downstream customers, and starch factories. Secondly, the platform coordinates the supply and demand sides to reach a cooperative agreement in the form of orders [70]. Finally, farmers are only required to plant high-quality products in accordance with the variety and quantity specified in the order, eliminating the need for them to worry about sales. The platform’s sales guarantee model, represented by the dashed box in Figure 9, provides a viable solution to the issue of overproduction. In the event of overproduction or late sales, the platform collaborates with starch factories to purchase the surplus produce at cost price.

### 4.4. Capabilities Generated from the Integration of the Three Flows

#### 4.4.1. Inventory Financing

Inventory financing is a subset of supply chain financing [71]. Agricultural supply chains (ASCs) are often characterized by seasonal harvesting, resulting in a large capital demand during the raw material procurement period, but a long payback period for product sales, leading to a funding gap. To address this issue, Tudouec integrated the advantages of information flow, bank capital flow, and material flow to create a potato warehouse receipt pledge financing model.

**Figure 10:**
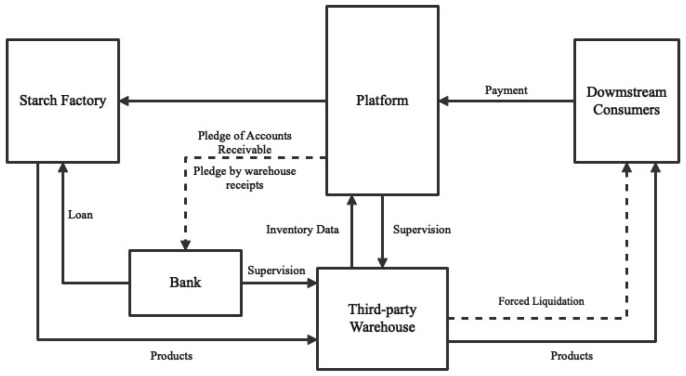
Inventory financing model.

The operation process of this model is illustrated in Figure 10. The first step involves a joint pre-lending investigation, conducted by the bank and the platform, followed by the provision of loans to the starch factory, with the inventory as collateral. Secondly, the starch factory and the platform share transaction data related to the raw material warehouse, the starch warehouse, and the starch. The pledged goods are stored in an intelligent warehouse that is supervised by the starch factory, the bank, and the platform, with real-time inbound and outbound information being synchronized to the platform. Lastly, the starch factory sales are settled through the platform, enabling monitoring of accounts receivable. In the event of loan delinquency or other repayment difficulties, the platform can repay the loan using the accounts receivable, or liquidate the starch in stock at the current price to repay the loan.

#### 4.4.2. Risk Control and Management

Compared to other supply chains, the Agricultural Supply Chain (ASC) is subject to a higher number of sources of uncertainty and risk, particularly when compared to a traditional model where the risks are primarily borne by farmers [65]. The integration of three streams of information, capital, and material has led to the development of a systematic supply chain risk management system, which effectively minimizes the risks faced by each participant. For farmers, the platform’s implementation of order-based sales addresses the concern of marketing, while supply chain financing addresses the risk of insufficient capital. Insurance further protects farmers from financial loss due to natural disasters. The platform also facilitates quality control and price negotiation through bulk purchasing of high-quality and cost-effective agricultural products and mechanized equipment, thereby reducing the price of fertilizers and equipment and ensuring the quality of agricultural materials. Insurance institutions can benefit from the platform’s sharing of planting data, which enables real-time tracking and monitoring and reduces regulatory costs, thereby minimizing moral hazards. Financial institutions can benefit from the platform’s provision of transaction information, such as orders, warehousing, logistics, and more, ensuring the authenticity of trade. Risk mitigation measures, including pledges of accounts receivable, help guarantee the source of repayment, and third-party intelligent warehousing supervision ensures the validity of inventory pledges. The forced liquidation mechanism further protects funds in the event of a market downside risk. For starch factories, the platform reduces the risks associated with raw material sourcing and helps to control the quality and variety of raw materials, while also addressing the risk of insufficient capital and sales. However, it is important to note that the platform, as an intermediary and information manager, can cause significant harm to all participants if moral risks or information security breaches occur. As a result, constraints and security mechanisms are necessary to monitor and ensure the stable operation of the platform.

### 4.5. Effect of the Integration of the Three Flows

The integration of the three flows has led to the development of a new supply chain model, which optimizes the SCM level and enhances the support for real agriculture. The platform model effectively addresses the issues faced by traditional agriculture in regards to marketing, access to information, financing, storage, and transportation (as outlined in Table 4). By combining capital flow, information flow, and material flow, the platform exhibits a robust supply chain service capability. From an SCM perspective, the platform ensures the seamless transportation of products, efficient dissemination of information, and secure financial transactions. The e-commerce platform serves as the central component of the supply chain, connecting all participants in the model, and can be viewed as a centralized information platform, due to the collection, integration, and sharing of information within the system. The efficient operation of the information flow removes the obstacles previously faced by capital flow and logistics operations. By establishing a comprehensive network of material flow, capital flow, and information flow, barriers from supply to demand are lifted, and new ASC management tools, such as supply chain financial services, insurance services, logistics, and warehousing services, are created, greatly improving the efficiency of the overall supply chain operations.

The traditional intermediary modes of brokers take advantage of information asymmetry, but the platform-oriented SCM mode promotes information interaction among the participants and weaves the whole supply chain network using the information as the hub. The interaction of information flow with material and capital flows enables supply chain services widely used in industry, such as supply chain finance, intelligent storage, and intelligent logistics, to be applied in the agricultural field. Therefore, rather than calling the e-commerce platform model an “internet+agriculture” business model, it is better to call it an information-based SCM model. On one hand, the e-commerce platform provides high-quality supply chain services through the interconnection of information technology. On the other hand, the optimization of the supply chain by the e-commerce platform enhances SCM capability.

## 5. Conclusions

### 5.1. Theoretical Contributions

This paper contributes to the field of SCM literature by making three theoretical contributions. First, it seeks to define the relationship between information flow, material flow, and capital flow by using the case study of Tudouec. The paper finds that Tudouec’s efficient information flow system improves the efficiency of both material and capital flow. Second, this study is the first to examine the resources embedded in the three processes, and to identify the resulting supply chain capabilities. It proposes that the e-commerce platform’s enhanced supply chain service capability comes from the interaction and combination of these processes. Third, the paper introduces the concept of using an e-commerce platform to optimize SCM and service capabilities. This platform is seen as an ideal environment to gain insight into the supply chain concept, and research suggests that the core competency of agricultural e-commerce platforms is providing high-quality supply chain services by managing these three flows.

### 5.2. Managerial Contributions

The findings of this study offer valuable managerial insights into the potential impact of an agricultural e-commerce platform on rural development. The ASC e-commerce platform studied here demonstrated its ability to reduce the risk of the agricultural supply chain, improve the quality of agricultural products, and promote collaboration among stakeholders. By converging three streams, the platform created a more efficient and effective system for the entire agricultural product life cycle, including production, processing, distribution, and financing. This led to not only reduced risk in the agricultural supply chain, but also improved quality and competitiveness of farmers and the sector as a whole. Additionally, the platform facilitated cooperation and mutual benefits among farmers, distributors, and consumers. These insights emphasize the importance of leveraging technology and innovative solutions in addressing the challenges faced by the agricultural industry, and highlight the potential for the platform to facilitate rural revitalization. This study contributes to the existing literature that supports the viability of rural e-commerce for promoting and developing various agricultural products in developing countries [72,73,74]. As a mature e-commerce platform for potatoes, Tudouec showcases a successful business model and supply chain management approach that has the potential to be replicated and adapted for other agricultural contexts. Overall, this study has important implications for rural management.

### 5.3. Limitation and Future Research Directions

This study was limited in several ways that should be addressed in future research. Firstly, the sample size of the present study was limited to a single entity, Tudouec. Further research is needed to expand the sample size and conduct multiple case studies. Secondly, the time period of this study was relatively short, so may not provide a comprehensive picture of the situation. Longitudinal studies with a longer time frame are required to better understand the long-term operation and development of the platform. Thirdly, this study mainly employed a qualitative research approach, leading to more subjective findings. Future research should aim to increase the level of objectivity by incorporating quantitative methods. Fourthly, the focus of this study was on a potato e-commerce platform in the Inner Mongolia region. Research in different geographical and agricultural contexts is needed to generalize the results. Additionally, future research should explore various types of rural e-commerce platforms not covered in this study and compare the results with those presented in this paper.

## Figures and Tables

**Table 1:** Composition of the Tudouec Platform.

Abbreviation	Full Company Name	Establish Date	Location	Main Business Area
Jinke	Inner Mongolia Jinke Investment Management Co., Ltd.	2010	Inner Mongolia	Information platform and online trading platform
Goden Bean	Inner Mongolia Goden Bean Agircultural Technology Co., Ltd.	2014	Inner Mongolia	Site supervision and technical guidance
Tengzhou	Tengzhou Potato E-commerce Trading Co., Ltd.	2014	Shandong Province	Offline sales, agricultural materials procurement, and mechanized equipment procurement

**Table 2:** List of interviews.

Title	Number of Interviews	Interview Method
CEO of Tudouec	1	Face-to-face
Chief financial officer of Tudouec	1	Face-to-face
Technical director of Tudouec	3	Face-to-face
Salesman from Tudouec	5	Telephone
Potato farmer representatives	10	Face-to-face
Business manager of an insurance company	2	Face-to-face
Vice president of a financial institution	1	Face-to-face
Representative of a seeds supplier	1	Telephone
Representative of a fertilizer supplier	1	Telephone
Starch factory managers	2	Face-to-face
Rural villagers (not involved with Tudouec)	5	Face-to-face

**Table 3:** The current situation of the traditional agricultural model and the problems faced by farmers.

Categories	The Current Situation of Traditional Agriculture	Problems
Marketing channel	1. Local retail	1. Limited sales channel
	2. Brokers’ purchase	2. Low bargaining capability
	3. Starch factories’ purchase	
Information acquisition	Information isolation and asymmetry	1. Unable to obtain market information and price fluctuation trend
		2. Difficult to adjust the planting, storage, and sales scales in time
		3. Lack of analysis and data utilization
Planting	1. Experience-based planting	1. Difficult to implement new breeds and new planting skills
	2. Labor-intensive farming	2. Low mechanical cultivation
	3. Individual planting without an industry leader and organization	3. Lack of planting technical support
		4. Lack of standardization and brand
Financing	1. Limited channels to obtain financial support	1. Unable to expand their production scale
	2. Lack of valid collateral	2. Unable to finance through trade
	3. Payment source unstable	
Insurance	1. High insurance premiums	1. Weak risk resistibility
	2. Difficult to claim	2. Have actual insurance requirement but unable to afford
Logistics	1. Bulk transport	1. High loss in transportation
	2. Short-distance transportation	2. Products cannot be sold to a faraway location
	3. Low coverage of cold chain transport	3. Hardly able to track
	4. High transportation costs	
	5. No tracking and tracing system	
Storage	1. Limited storage space, inadequate infrastructure, inadequate ventilation, and difficulty in regulating temperature and humidity.	Improper storage causes decay, sprouting, water loss, frost damage, greening, and other problems, leading to loss of commercial value and of food value
	2. Refrigerated warehouses are rare and expensive	

**Table 4:** Comparison of models based on the farmers’ perspective.

	Traditional Model	Tudouec Model
Sales channel	Brokers/Starch factory/Local Sale	Platform Trading/Order Selling/Starch factory/Local sale
Information acquisition ability	Weak	Strong
Production	Unstable	Increase
Financing capacity	Limited	Strong
Agricultural insurance	Rare	Popular
Risk	High	Low
Warehousing	Self-built, poor condition	Professional warehousing
Transport	High loss and high price	Low loss and low price
Revenue	High volatility	Stability

## Data Availability

The data presented in this study are available within the article.
